# A New and Efficient Synthesis of 6-*O*-Methylscutellarein, the Major Metabolite of the Natural Medicine Scutellarin

**DOI:** 10.3390/molecules200610184

**Published:** 2015-06-02

**Authors:** Wei Zhang, Ze-Xi Dong, Ting Gu, Nian-Guang Li, Peng-Xuan Zhang, Wen-Yu Wu, Shao-Peng Yu, Yu-Ping Tang, Jian-Ping Yang, Zhi-Hao Shi

**Affiliations:** 1 National and Local Collaborative Engineering Center of Chinese Medicinal Resources, Industrialization and Formulae Innovative Medicine, Nanjing University of Chinese Medicine, Nanjing 210023, China; E-Mails: zhangwei20131405@163.com (W.Z.); dongzexi1215@163.com (Z.-X.D.); guting1992@163.com (T.G.); zpx20130901@126.com (P.-X.Z.); 15150513147@163.com (W.-Y.W.); yushaopeng0405@163.com (S.-P.Y.); yupingtang@njutcm.edu.cn (Y.-P.T.); jpyang-0828@163.com (J.-P.Y.); 2 Department of Organic Chemistry, China Pharmaceutical University, Nanjing 211198, China

**Keywords:** scutellarin, scutellarein, 6-*O*-methylscutellarein, metabolite, synthesis

## Abstract

In this paper, a new and efficient synthesis of 6-*O*-methylscutellarein (**3**), the major metabolite of the natural medicine scutellarin, is reported. Two hydroxyl groups at C-4′ and C-7 in **2** were selectively protected by chloromethyl methyl ether after the reaction conditions were optimized, then 6-*O*-methyl-scutellarein (**3**) was produced in high yield after methylation of the hydroxyl group at C-6 and subsequent deprotection of the two methyl ether groups.

## 1. Introduction

As a frequently-occurring disease that seriously endangers human health, ischemic cerebrovascular disease is one of the leading causes of disability and death worldwide [1]. The critical role that thrombin plays in ischemic cerebrovascular disease has been suggested by increasing evidence [2] that thrombin catalyzes the proteolytic cleavage of the soluble plasma-protein fibrinogen to form insoluble fibrin leading to clot formation [3]. Furthermore, the excessive production of reactive oxygen species (ROS) [4,5] including the superoxide anion radical (O_2_^−•^), hydroxyl radical (^•^OH), hydrogen peroxide (H_2_O_2_), singlet oxygen (^1^O_2_), and nitric oxide (NO^•^) may result in increased levels of low-density lipoprotein (LDL), oxidative modification of LDL, and an impairment of endothelial derived relaxing factor (EDRF, nitric oxide, NO)-mediated bioactions [6], thus causing ischemic cerebrovascular disease.

**Figure 1:**
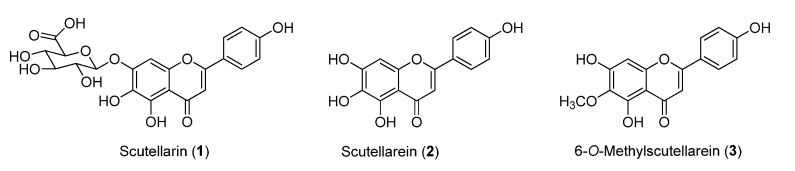
Chemical structures of scutellarin (**1**), scutellarein (**2**) and 6-*O*-methylscutellarein (**3**).

Some natural products with anticoagulant capacity and antioxidant activity have been proposed to treat ischemic cerebrovascular disease. Traditional Chinese Medicines can be regarded as potential rich sources for the discovery of drug lead compounds because they have been used clinically for thousands years. Breviscapine, which is a natural drug consisting of total flavonoids of *Erigeron breviscapus* (Vant.) Hand-Mazz. (Compositae), has a large market share in China for the treatment of angina pectoris, cerebral infarction and coronary heart disease [7]. Pharmacological studies have found that scutellarin (**1**, Figure 1), the main effective constituent (>85%) of breviscapine, exhibited anticoagulant and antioxidant activities that attenuate neuronal damage, and thus has a wide range of benefits for brain injury caused by cerebral ischemia/reperfusion [8,9,10].

Interestingly, pharmacokinetic studies showed that the oral bioavailability of scutellarin (**1**) was quite poor [11] in humans [12], because scutellarin (**1**) is hydrolyzed to scutellarein (**2**, Figure 1) before absorption, and scutellarein (**2**) can metabolize into methylated, sulfated or glucuronidated forms [13] in the blood. 6-*O*-Methylscutellarein (**3**, Figure 1), which is one of the major *in vivo* metabolites might be responsible for the therapeutic effects of scutellarin (**1**), however, this interesting scutellarin metabolite is not commercially available, so the chemical synthesis of 6-*O*-methyl-scutellarein (**3**) [14] is very important for further study.

**Scheme 1:**
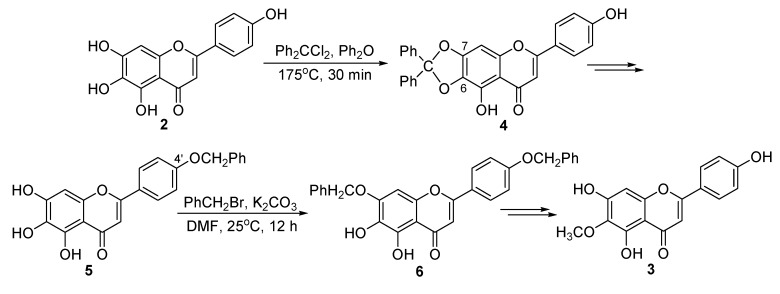
The previous synthetic route of 6-*O*-methylscutellarein (**3**).

Recently, we reported a synthetic route (Scheme 1) for the construction of 6-*O*-methylscutellarein (**3**) [15]. Dichlorodiphenylmethane was used first to protect the dihydroxyl groups at C-6 and C-7 in scutellarein (**2**). Subsequently, benzyl bromide was used to selectively protect the hydroxyl groups at C-4′ in **4** and at C-7 in **5**, respectively. Finally, 6-*O*-methylscutellarein (**3**) was obtained after methylation at C-6 in **6** followed by deprotection of the two benzyl groups at C-7 and C-4′.

However, the above reaction conditions using dichlorodiphenylmethane at 175 °C to protect the dihydroxy groups at C-6 and C-7 in scutellarein (**2**) would hamper the large-scale preparation of 6-*O*-methylscutellarein (**3**). After an extensive study, we optimized the reaction conditions and used the chloromethyl methyl ether to directly protect the hydroxyl groups at C-4′ and C-7 in **2**, so 6-*O*-methyl-scutellarein (**3**) was obtained in high yield in only four steps from scutellarin (**1**). Herein, we report the details of this new synthetic route.

## 2. Results and Discussion

**Scheme 2:**
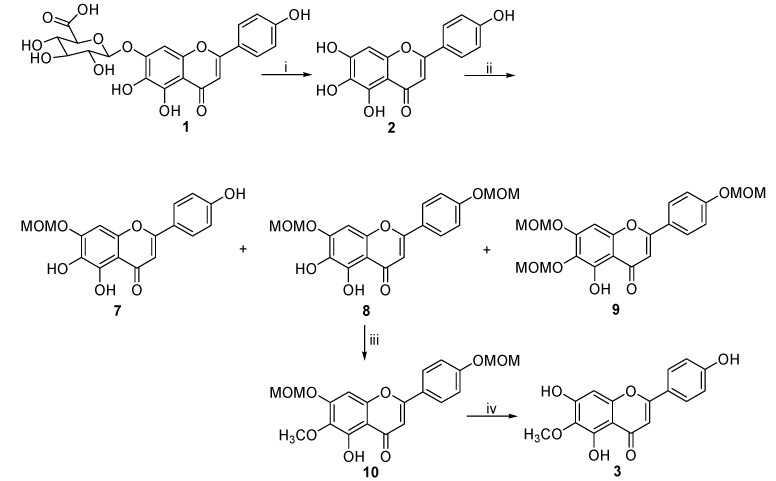
The new synthetic route of 6-*O*-methylscutellarein (**3**).

As shown in Scheme 2, scutellarein (**2**) was obtained from the hydrolysis of scutellarin (**1**) by refluxing it with 6 N HCl in 90% ethanol under the N_2_ protection [16,17]. Based on the different reactivity of the four hydroxyl groups in scutellarein (**2**) which follows the specific sequential position order 7 > 4ʹ > 6 > 5, the direct alkylation of scutellarein (**2**) with chloromethyl ether should lead mainly to the formation of disubstituted product **8**. Firstly, the treatment of **2** with 4.0 equiv. of chloromethyl ether and 4.2 equiv. K_2_CO_3_ in acetone under reflux afforded three products **7**, **8** and **9** in 14.8%, 28.9% and 34.2%, repectively. The location of MOM groups in these three compounds was confirmed by ^1^H-NMR and ROESY. In the ^1^H-NMR (in DMSO-*d*_6_) spectrum of **7**, a signal at δ 12.40 suggested that 5-OH remained unchanged, and a cross-peak observed in the ROESY spectrum of **7** between 5.35 (-OCH_2_) with 6.66 (8-H) indicated that the only one MOM group was at the C7 position. In the ^1^H-NMR spectrum of **8** (in DMSO-*d*_6_), a signal at δ 12.76 indicated that the 5-OH group remained unchanged. One cross-peak observed in the ROESY spectrum of **8** between 5.35 (-OCH_2_) with 6.69 (8-H) indicating that one of the two MOM groups was at C7, while another cross-peak between 5.22 (-OCH_2_) with 7.05 (3′,5′-H) indicated that the second MOM group was at C4′. In the ^1^H-NMR spectrum of **9** (in DMSO-*d*_6_), a signal at δ 12.93 indicated that the 5-OH group remained unchanged, and three signals at δ 5.35, 5.48 and 5.60 showed that three MOM groups had been substituted at the C4′, C6 and C7 positions.

In order to improve the yield of the target compound **8**, we subsequently optimized this reaction conditions by changing the ratio of chloromethyl ether. When the amount of chloromethyl ether was increased to 4.2 equiv. (Table 1, Run 2) and 4.4 equiv. (Table 1, Run 3), the yield of **8** was reduced to 24.3% and 21.6%, and the yield of the side product **9** was increased to 38.7% and 41.8%, respectively. This result indicated that the ratio of chloromethyl ether should be decreased. As shown in Run 4, the yield of **8** increased to 32.1% when the ratio of chloromethyl ether was set at 3.8 equiv. Furthermore, the yield of **8** could be increased to 43.2% (Table 1, Run 6) after **2** reacted with 3.4 equiv. of MOMCl and 3.6 equiv. of K_2_CO_3_ under reflux in acetone. Unfortunately, the yield of the target compound **8** decreased to 38.4% and 36.2% and that of the side product **7** increased to 20.6% and 21.3% when the amount of chloromethyl ether was decreased to 3.4 equiv. and 3.2 equiv. respectively, so the best reaction conditions for the synthesis of the target compound **8** were a ratio of MOMCl and K_2_CO_3_ of 3.4 equiv. and 3.6 equiv. respectively.

**Table 1:** Optimization of reaction conditions in the synthesis of **8**.

Run	Reaction Conditions	Yield (%)
1	MOMCl (4.0 equiv.), K_2_CO_3_ (4.2 equiv.), acetone, reflux, N_2_, 8 h	**7** (14.8), **8** (28.9), **9** (34.2)
2	MOMCl (4.2 equiv.), K_2_CO_3_ (4.4 equiv.), acetone, reflux, N_2_, 8 h	**7** (12.1), **8** (24.3), **9** (38.7)
3	MOMCl (4.4 equiv.), K_2_CO_3_ (4.6 equiv.), acetone, reflux, N_2_, 8 h	**7** (10.2), **8** (21.6), **9** (41.8)
4	MOMCl (3.8 equiv.), K_2_CO_3_ (4.0 equiv.), acetone, reflux, N_2_, 8 h	**7** (16.4), **8** (32.1), **9** (31.6)
5	MOMCl (3.6 equiv.), K_2_CO_3_ (3.8 equiv.), acetone, reflux, N_2_, 8 h	**7** (18.2), **8** (35.8), **9** (26.5)
6	MOMCl (3.4 equiv.), K_2_CO_3_ (3.6 equiv.), acetone, reflux, N_2_, 8 h	**7** (19.2), **8** (43.2), **9** (18.7)
7	MOMCl (3.2 equiv.), K_2_CO_3_ (3.4 equiv.), acetone, reflux, N_2_, 8 h	**7** (20.6), **8** (38.4), **9** (16.4)
8	MOMCl (3.0 equiv.), K_2_CO_3_ (3.2 equiv.), acetone, reflux, N_2_, 8 h	**7** (21.3), **8** (36.2), **9** (14.2)

*Reagents and conditions*: (i) HCl, EtOH, N_2_, reflux, 36 h, 17%; (ii) MOMCl (3.4 equiv.), K_2_CO_3_ (3.6 equiv.), acetone, reflux; (iii) CH_3_I (1.2 equiv.), K_2_CO_3_ (1.4 equiv.), DMF, 25 °C, 6 h, 94%; (iv) HCl (1.0 M) in Et_2_O/CH_2_Cl_2_ (1:1), 25 °C, 1 h, 92%.

With 4ʹ,7-dimethoxymethylated scutellarein (**8**) in hand, 6-*O*-methylscutellarein (**3**) was synthesized according to our previous procedure [15]. The reaction of **8** with 1.2 equiv. of iodomethane led selectively to **10** with the desired methyl group on the C6-OH in 94% yield. Then the deprotection of di-MOM groups under hydrolysis conditions in Et_2_O/CH_2_Cl_2_ using hydrochloric acid as the catalyst afforded **3** in 92% yield.

In summary, we have developed a new and efficient process for the synthesis of 6-*O*-methyl-scutellarein (**3**) in only four steps. Based on the fact the relative activity of the four hydroxyl groups is different in the order 7 > 4′ > 6 > 5, chloromethyl methyl ether was used to protect the hydroxyl groups at C-4′ and C-7 in **2** after the reaction conditions were optimized, then 6-*O*-methylscutellarein (**3**) was produced in high yield after methylation of the hydroxyl group at C-6 and deprotection of the two methyl ether groups. This synthetic procedure is very efficient and effective and could be applied to the selective synthesis of other *O*-methylflavonoid isomers as well as the other flavonoid sulfate- and glucuronide-metabolites *in vivo*.

## 3. Experimental Section

### General Information

Reagents and solvents were purchased from commercial sources and used without further purification unless otherwise specified. Air- and moisture-sensitive liquids and solutions were transferred via syringe or stainless steel cannula. Organic solutions were concentrated by rotary evaporation below 45 °C at approximately 20 mm Hg. All non-aqueous reactions were carried out under anhydrous conditions using flame-dried glassware in nitrogen atmosphere in dry, freshly distilled solvents, unless otherwise noted. Yields refer to chromatographically and spectroscopically (^1^H-NMR) homogeneous materials, unless otherwise stated. Reactions were monitored by thin-layer chromatography (TLC) carried out on 0.15–0.20 mm silica gel plates (RSGF 254, Yantai, China) using UV light as the visualizing agent. Chromatography was performed on silica gel (160–200 mesh, Qingdao, China) with petroleum ether (60–90) and ethyl acetate mixtures as eluant. ^1^H-NMR spectra were obtained with an AV-300 (300 MHz) instrument (Bruker, Karlsruhe, Germany) Chemical shifts are recorded in ppm downfield from tetramethylsilane. *J* values are given in Hz. Abbreviations used are s (singlet), d (doublet), t (triplet), q (quartet), b (broad) and m (multiplet). ESI-MS spectra were recorded on a Synapt HDMS spectrometer (Waters, Milford, MA, USA).

*5*,*6*,*7-Trihydroxy-2-(4-hydroxyphenyl)-4H-chromen-4-one* (**2**). To a stirring mixture of **1** (10.0 g, 21.6 mmol) and concentrated hydrochloric acid (120 mL) in ethanol (120 mL) was added water (10 mL), and the reaction mixture was refluxed under a N_2_ atmosphere for 36 h. After cooling down to room temperature, the mixture was poured into water. The solid obtained was filtered followed by silica gel column chromatography purification of the residue using 50% ethyl acetate in petroleum ether to afford compound **2** (1.05 g, 17.0% yield) as yellow solid. ^1^H-NMR (DMSO-*d*_6_) δ 6.57 (s, 1H, 8-H), 6.74 (s, 1H, 3-H), 6.91 (d, *J* = 8.8 Hz, 2H, 3′,5′-H), 7.90 (d, *J* = 8.8 Hz, 2H, 2′,6′-H), 8.71 (s, 1H, 6-OH), 10.29 (s, 1H, 7-OH), 10.44 (s, 1H, 4′-OH), 12.78 (s, 1H, 5-OH); ESI-MS: *m*/*z* 287 [M + H]^+^.

*Synthesis of MOM substituted products* **7**, **8** *and* **9***.* To a stirring mixture of **2** (2 g, 7 mmol) and K_2_CO_3_ (3.48 g, 25.2 mmol, 3.6 equiv.) in dry acetone (100 mL) at room temperature was added chloromethyl ether (1.78 mL, 23.8 mmol, 3.4 equiv.), the reaction mixture was refluxed gently for 8 h. After cooling to room temperature, the reaction mixture was filtered. Removal of the solvent *in vacuo* followed by purification of the residue by column chromatography on silica gel with 20% ethyl acetate in petroleum ether as eluent afforded **7** (0.44 g, 19.2%), **8** (1.13 g, 43.2%) and **9** (0.55 g, 18.7%) as yellow solids, respectively.

*5*,*6-Dihydroxy-2-(4-hydroxyphenyl)-7-(methoxymethoxy)-4H-chromen-4-one* (**7**). ^1^H-NMR (DMSO-*d*_6_) δ 3.50 (s, 3H, -OCH_3_), 5.35 (s, 2H, -OCH_2_), 6.60 (s, 1H, 3-H), 6.66 (s, 1H, 8-H), 7.04 (d, 2H, *J* = 8.6 Hz, 3′,5′-H), 8.02 (d, 2H, *J* = 8.6 Hz, 2′,6′-H), 10.33 (s, 1H, 4′-OH), 12.40 (s, 1H, 5-OH); ESI-MS: *m/z* 329 [M − H]^−^.

*5,6-Dihydroxy-7-(methoxymethoxy)-2-(4-(methoxymethoxy)phenyl)-4H-chromen-4-one* (**8**). ^1^H-NMR (DMSO-*d*_6_) δ 3.52 (s, 3H, -OCH_3_), 3.61 (s, 3H, -OCH_3_), 5.22 (s, 2H, -OCH_2_), 5.35 (s, 2H, -OCH_2_), 6.61 (s, 1H, 3-H), 6.69 (s, 1H, 8-H), 7.05 (d, 2H, *J* = 8.6 Hz, 3′,5′-H), 8.01 (d, 2H, *J* = 8.6 Hz, 2′,6′-H), 12.76 (s, 1H, 5-OH); ESI-MS: *m/z* 373 [M − H]^−^.

*5-Hydroxy-6*,*7-bis(methoxymethoxy)-2-(4-(methoxymethoxy)phenyl)-4H-chromen-4-one* (**9**). ^1^H-NMR (DMSO-*d*_6_) δ 3.66 (s, 3H, -OCH_3_), 3.76 (s, 3H, -OCH_3_), 3.87 (s, 9H, -OCH_3_), 5.35 (s, 2H, -OCH_2_), 5.48 (s, 2H, -OCH_2_), 5.60 (s, 2H, -OCH_2_), 6.95 (s, 1H, 3-H), 7.09 (s, 1H, 8-H), 7.15 (d, 2H, *J* = 8.7 Hz, 3′,5′-H), 8.06 (d, 2H, *J* = 8.7 Hz, 2′,6′-H), 12.93 (s, 1H, 5-OH); ESI-MS: *m/z* 419 [M + H]^+^.

*5-Hydroxy-6-methoxy-7-(methoxymethoxy)-2-(4-(methoxymethoxy)phenyl)-4H-chromen-4-one* (**10**). To a stirring solution of **8** (67 mg, 0.18 mmol) in dry DMF (20 mL) was added K_2_CO_3_ (48 mg, 0.35 mmol, 1.4 equiv.) and iodomethane (0.014 mL, 0.22 mmol, 1.2 equiv.) at room temperature. After 12 h, the reaction mixture was then partitioned between ethyl acetate (100 mL) and water (100 mL). The ethyl acetate layer was then washed with brine (100 mL), dried over Na_2_SO_4_, filtered and concentrated. The crude material was purified by column chromatography (25% ethyl acetate in petroleum ether) to yield **10** (66 mg, 94% yield) as a yellow solid. ^1^H-NMR (DMSO-*d*_6_) δ ^1^H-NMR (300 MHz, DMSO-*d*_6_) δ 3.46 (s, 3H, -OCH_3_), 3.51 (s, 3H, -OCH_3_), 3.96 (s, 3H, -OCH_3_), 5.31 (s, 2H, -OCH_2_), 5.34 (s, 2H, -OCH_2_), 6.48 (s, 1H, 3-H), 6.62 (s, 1H, 8-H), 7.13 (d, 2H, *J* = 8.6 Hz, 3′,5′-H), 8.02 (d, 2H, *J* = 8.6 Hz, 2′,6′-H), 12.71 (s, 1H, 5-OH); ESI-MS: *m/z* 389 [M + H]^+^.

*5,7-Dihydroxy-2-(4-hydroxyphenyl)-6-methoxy-4H-chromen-4-one* (**3**). Hydrochloric acid (1 mL) was added to a stirred solution **10** (388 mg, 1 mmol) in CH_2_Cl_2_ (5 mL) and ether (5 mL) at 0 °C. The reaction mixture was allowed to warm to room temperature and stirred for a further 1 h. The reaction mixture was diluted with a large amount of ethyl acetate and washed with water and brine. The ethyl acetate layer was dried over MgSO_4_, filtered, then concentrated and the crude material purified by column chromatography (50% ethyl acetate in petroleum ether) to yield **3** (276 mg, 92%) as a yellow solid. ^1^H-NMR (DMSO-*d*_6_) δ 3.72 (s, 3H, -OCH_3_), 6.58 (s, 1H, 3-H), 6.89 (d, 2H, *J* = 8.6 Hz, 3′,5′-H), 7.11 (s, 1H, 8-H), 7.87 (d, 2H, *J* = 8.6 Hz, 2′,6′-H), 10.19 (s, 1H, 4′-OH), 10.72 (s, 1H, 7-OH), 12.76 (s, 1H, 5-OH); ESI-MS: *m/z* 299 [M − H]^−^.
